# Deep learning-based dose prediction in proton beam therapy for hepatocellular carcinoma: comparison of network architectures and loss functions

**DOI:** 10.1093/jrr/rrag026

**Published:** 2026-05-06

**Authors:** Shuta Ogawa, Noriyuki Kadoya, Takahiro Kato, Ryohei Kato, Ryota Tozuka, Yuki Narita, Sho Oyama, Masao Murakami, Keiichi Jingu

**Affiliations:** Department of Radiation Physics and Technology, Southern Tohoku Proton Therapy Center, 7-172 Yatsuyamada, Koriyama, Fukushima, 963-8052, Japan; Department of Radiation Oncology, Tohoku University Graduate School of Medicine, 1-1 Seiryou-machi, Aoba-ku, Sendai, Miyagi, 980-8574, Japan; Department of Radiation Oncology, Tohoku University Graduate School of Medicine, 1-1 Seiryou-machi, Aoba-ku, Sendai, Miyagi, 980-8574, Japan; Department of Radiological Sciences, School of Health Sciences, Fukushima Medical University, 10-6 Sakaemachi, Fukushima, Fukushima, 960-8516, Japan; Department of Radiation Physics and Technology, Southern Tohoku Proton Therapy Center, 7-172 Yatsuyamada, Koriyama, Fukushima, 963-8052, Japan; Department of Radiation Oncology, Tohoku University Graduate School of Medicine, 1-1 Seiryou-machi, Aoba-ku, Sendai, Miyagi, 980-8574, Japan; Department of Radiation Oncology, Tohoku University Graduate School of Medicine, 1-1 Seiryou-machi, Aoba-ku, Sendai, Miyagi, 980-8574, Japan; Department of Therapeutic Radiology, University of Yamanashi, 1110 Shimokato, Chuo-city, Yamanashi, 409-3898, Japan; Department of Radiation Physics and Technology, Southern Tohoku Proton Therapy Center, 7-172 Yatsuyamada, Koriyama, Fukushima, 963-8052, Japan; Department of Radiation Physics and Technology, Southern Tohoku Proton Therapy Center, 7-172 Yatsuyamada, Koriyama, Fukushima, 963-8052, Japan; Department of Radiation Oncology, Southern Tohoku Proton Therapy Center, 7-172 Yatsuyamada, Koriyama, Fukushima, 963-8052, Japan; Department of Radiation Oncology, Tohoku University Graduate School of Medicine, 1-1 Seiryou-machi, Aoba-ku, Sendai, Miyagi, 980-8574, Japan

**Keywords:** proton beam therapy, deep learning, dose prediction, hepatocellular carcinoma

## Abstract

In proton beam therapy (PBT) for hepatocellular carcinoma (HCC), deep learning (DL)-based dose prediction offers clinical value by providing immediate reference dose distributions as a guideline tool for treatment planners and enabling virtual PBT dose assessment at institutions lacking PBT facilities to support clinical decision-making. This study proposes a dose gradient-aware DL training approach and a beam arrangement-free framework that predicts dose distributions from computed tomography (CT) images and target/organs-at-risk structures without requiring beam arrangement inputs. Using data from 172 HCC patients, we systematically compared 20 DL models combining five architectures with four loss functions, including a novel dose gradient-aware loss capturing PBT-specific dose distribution characteristics arising from the Bragg peak. Prediction accuracy was evaluated using dose metrics for clinical target volume, planning target volume, and normal liver, 10% dose region volume assessment, visual examination, Dice similarity coefficient (DSC) for 10% dose region, and dose–volume histogram (DVH) analysis. DL models employing dose gradient-aware loss demonstrated reduced prediction errors in target volume dose metrics, achieved the lowest errors in 10% dose region volume when combined with U-Net (MAE: 35.4 cm^3^, RMSE: 51.3 cm^3^), attained the highest DSC (0.88 ± 0.05), showed the closest DVH agreement with clinical plans for both target structures and normal liver. A comprehensive evaluation consistently confirmed the superiority of dose gradient-aware loss across all metrics, demonstrating the importance of physics-informed, dose gradient-aware learning for accurate PBT dose distribution prediction. Particularly, DL models combining simple architectures with dose gradient-aware loss predicted doses that closely approximated clinical doses in PBT.

## INTRODUCTION

In 2022, liver cancer was globally ranked as the sixth most frequently diagnosed cancer and the third leading cause of cancer mortality, with hepatocellular carcinoma (HCC) accounting for 75–85% of cases [1]. Survival rates for HCC are low, with a relative 5-year overall survival rate of ~18% [2]. Proton beam therapy (PBT) is the treatment of choice as a useful treatment option for HCC. It is characterized by the Bragg peak, a physical property of proton beams that allows a considerable reduction in healthy tissue radiation exposure compared with photon beam therapy [3–9]. PBT for HCC has also been shown to significantly reduce the risk of radiation-induced liver injury, resulting in marked increases in overall survival rates [6]. Thus, PBT may substantially improve prognosis in patients with HCC.

Several uncertainties exist in treatment planning for PBT. Due to the range estimation uncertainties and anatomical variations, such as respiratory motion, dose distributions in PBT are more prone to degradation than those for photon beam therapy [10–13]. The strategic selection of optimal beam arrangements is essential to mitigate these uncertainties and minimize dose distribution degradation [14–16]. In PBT for HCC, beam arrangements are selected based on tumor location and size, the anatomical configuration of the surrounding healthy tissue, and the differential respiratory motion observed across liver segments [17]. These factors necessitate substantial time and effort in treatment planning. The quality of the resulting plan is highly dependent on the planner’s experience and expertise, and the time spent on the planning process.

Against this background, there is a growing demand for technical and operational improvements to the efficacy and quality of PBT. Consequently, research on the use of deep learning (DL) techniques in PBT treatment planning has intensified [14, 15, 18–30]. A notable approach has achieved accurate and efficient dose prediction by creating beam masks from pre-selected beam angles and numbers. The beam masks were then employed in conjunction with sliding window techniques to train a DL model focused on the local details of dose distributions [26]. Additionally, it has been demonstrated that combining beam masks with data aggregation significantly improves the accuracy of DL-based proton dose prediction in cases requiring complex treatment planning [29].

However, these previous approaches require beam masks based on predetermined beam arrangements, complicating the workflow of dose distribution prediction and introducing inherent limitations. Since beam arrangement selection critically influences plan quality, an inappropriate selection precludes clinically acceptable dose distributions. Furthermore, as the predicted dose distribution shape is constrained by the prepared beam masks, the DL model’s capacity to explore alternative beam arrangements is restricted. Consequently, when alternative beam arrangements are considered, the beam masks must be recreated and the model re-inferred, diminishing the flexibility of dose prediction process. Furthermore, these previous studies have only evaluated their prediction results with a single network architecture. None have proposed a suitable architecture for dose distribution prediction in PBT. Additionally, investigation into loss functions designed for PBT remains insufficient. Although the characteristic dose gradient patterns formed by the physical properties of the Bragg peak are of critical clinical importance in PBT, no loss function has yet been proposed to explicitly capture these gradient characteristics. These limitations constrain the applicability of DL techniques in clinical settings, for which a more comprehensive and practical approach is needed.

To address these limitations, this study developed DL models to predict dose distributions for PBT for HCC, with contributions in the following three aspects. First, we propose a beam arrangement-free approach that uses only computed tomography (CT) images and target/organs-at-risk (OAR) structures as input to directly predict the dose distribution that would result from an optimal beam arrangement, without requiring beam arrangement information. This approach offers a more flexible and accessible methodology that can serve as a guideline tool, providing treatment planners with immediately available dose distributions for reference during the early stages of treatment planning. Furthermore, this approach enables virtual evaluation of PBT dose distributions at institutions lacking PBT facilities or expertise, thereby supporting clinical decision-making regarding treatment options and assessment of treatment feasibility. Second, we propose a novel gradient-aware loss that evaluates direction-independent gradient magnitude in 3D dose distributions. Although gradient-based loss functions have been widely used in image processing, their application to dose distribution prediction in radiotherapy has not yet been reported. This loss function was designed to capture dose gradient patterns specific to PBT, enabling the DL model to implicitly learn beam paths through dose gradient patterns in the lateral and distal fall-off regions. Third, we constructed a total of 20 DL models by combining five network architectures with four loss functions, and systematically evaluated the accuracy of dose distribution prediction in PBT.

## MATERIALS AND METHODS

### Patient data

This study included data from 172 patients who underwent PBT between 2018 and 2024. Of these, 135 patients received 66 Gy (relative biological effectiveness [RBE]) in 10 fractions for primary HCC, while the remaining 37 received 64 Gy(RBE) in eight fractions for metastatic liver tumor [31, 32]. All patients fulfilled the eligibility criteria for the treatment protocols. The criteria required a distance of ≥20 mm between the tumor and at least one first-order branch or the main trunk of the portal vein, or between the tumor and the gastrointestinal tract including the duodenum [33]. The only differences between the two treatment protocols are the prescription dose and the number of fractions; the overall treatment planning policy is consistent for both. The collection and analysis of patient data for this study were conducted with the approval of our institutional review board (approval no. 599).

Treatment planning CT images were acquired using an Aquilion LB (Canon Medical Systems, Otawara, Japan). Patients were placed in the supine position with their arms over their heads and then immobilized using a vacuum cushion. The CT scan was performed with respiratory gating using a laser displacement sensor, AZ-733 V (Anzai Medical, Tokyo, Japan). The scans included the entire liver with a slice thickness of 2 mm. The treatment planning CT data were imported into the treatment planning system (TPS), XiO-M 4.34.02 (Hitachi, Kashiwa, Japan). Target volumes and OARs were delineated by experienced radiation oncologists. Clinical target volumes (CTVs) were defined by adding a 5 mm clinical margin to the gross tumor volume (GTV). Planning target volumes (PTVs) were established by applying additional safety margins of 5 mm in the anterior–posterior and right–left directions, and of 7 mm in the superior–inferior direction, to account for respiratory motion.

### Treatment planning

Treatment plans were created using XiO-M software and were based on the beam data of the passive scattering (PS) PBT system, Melthea (Hitachi, Kashiwa, Japan). The dose distributions were calculated as 2 × 2 × 2 mm grids. The calculation algorithm used in the TPS was a commercial pencil beam algorithm [34]. For all cases, the dose distributions were normalized such that the prescribed dose was delivered to 50% of the CTV. This dose normalization approach was selected to reduce inter-patient variability in dose distributions, particularly because the patient cohort included cases where the CTV was located close to OARs such as the ribs.

The irradiation technique used was a PS method known as the wobbler technique. Beam energy of either 150 or 210 MeV was selected based on tumor location and size. The PTV was established as the irradiation target area, and treatment was delivered using irradiation fields shaped by a multi-leaf collimator (MLC). An MLC margin of 7 mm was applied relative to the PTV. Patients were placed in the supine position during treatment, and two beams were used. The optimal beam angles were selected from 71 possible options at 5° intervals. The beam parameters were determined by experienced medical physicists, and the final dose distribution underwent a secondary check by a different medical physicist to minimize any planner bias.

### Deep learning architectures

In this study, five types of network architecture (U-Net, dense dilated U-Net [DD U-Net], hierarchically densely connected U-Net [HD U-Net], no-new-Net [nnU-Net], and UNEt TRansformers [UNETR]) were used for dose distribution prediction. These network architectures have demonstrated high accuracy in dose distribution prediction for photon beam therapy [18, 35–37]. These architectures were selected because they exhibit different feature extraction capabilities and spatial information preservation properties. This selection enabled systematic investigation of which architecture, when combined with different loss functions, most effectively captures the dosimetric characteristics of PBT, including the steep dose gradient patterns inherent to proton beams. They utilize input from treatment planning CT images and target/OAR structures to predict 3D dose distribution.

U-Net employs a basic encoder-decoder structure with skip connections [38]. DD U-Net extends U-Net by integrating dense connections and dilated convolutions [35], while HD U-Net combines the strengths of U-Net and DenseNet architectures [36]. The DD U-Net and HD U-Net architectures were reproduced and used under the same conditions as those described in previous studies [35, 36]. nnU-Net is a self-configuring framework that automatically optimizes the entire pipeline for medical image segmentation tasks [39]. UNETR utilizes a Vision Transformer as an encoder and a convolutional neural network as a decoder [40].

### Loss functions

Four types of loss functions were used in our DL model training. The mean squared error (MSE) loss and the weighted mean squared error (wMSE) loss, both based on region of interest (ROI), were selected as they are commonly used to predict dose distributions in photon beam therapy [35–37, 41]. Additionally, we developed two novel loss functions: one based on a dose–volume histogram (DVH loss) and another that utilizes dose gradient distribution (dose gradient loss). The four loss functions were combined in a complementary manner to capture proton dose distribution characteristics. We will now describe each loss function in detail.

#### MSE loss

MSE loss is a standard means of evaluating the discrepancy between the clinical dose distribution (cliDose) and the predicted dose distribution (preDose). The formula for MSE loss is as follows:


(1) \begin{equation*} \mathrm{MSE}\ \mathrm{loss}=\frac{1}{\mathrm{N}}\sum_{\mathrm{i}=1}^{\mathrm{N}}{\left({\mathrm{preDose}}_{\mathrm{i}}-{\mathrm{cliDose}}_{\mathrm{i}}\right)}^2 \end{equation*}

where $\mathrm{N}$ is the total number of voxels in the dose distribution, ${\mathrm{preDose}}_{\mathrm{i}}$ is the predicted dose, and ${\mathrm{cliDose}}_{\mathrm{i}}$ is the clinical dose.

#### wMSE loss

The wMSE loss is an extension of the standard MSE loss in which prediction errors are calculated with different weights assigned to each target/OAR structure [35]. The weighting factor for the CTV was set to 1.0, while the weights for the PTV, liver, spinal cord, and intestines were assigned values of 0.8, 0.5, 0.2, and 0.2, respectively. These weighting factors reflect the dose constraint prioritization of structures at our institution. Hence, the highest weight is assigned to the CTV as it has the highest priority in dose constraint considerations. The weights for the other structures were set based on the relative importance of their dose constraints during clinical treatment planning. The formula for the wMSE loss is as follows:


(2) \begin{equation*} \mathrm{wMSE}\ \mathrm{loss}=\sum_{\mathrm{ROI}\mathrm{s}}{\mathrm{w}}_{\mathrm{ROI}}^2\cdot \frac{1}{{\mathrm{N}}_{\mathrm{ROI}}}\sum_{\kern0.5em \mathrm{i}=1}^{{\mathrm{N}}_{\mathrm{ROI}}}{\left({\mathrm{preDose}}_{\mathrm{i}}-{\mathrm{cliDose}}_{\mathrm{i}}\right)}^2 \end{equation*}

where ${\mathrm{w}}_{\mathrm{ROI}}$ is the weight assigned to each target structure or OAR, and ${\mathrm{N}}_{\mathrm{ROI}}$ is the total number of voxels within the dose distribution for each respective target structure or OAR.

#### DVH loss

DVH loss_CTV_ evaluates the dose values based on D_98%_ and D_2%_ for the CTV. These represent the doses covering 98% and 2% of the target volume, respectively. For the normal liver (defined as the liver volume excluding the GTV), DVH loss_normal liver_ assesses the volume percentages receiving >30 Gy(RBE) (V_30 Gy(RBE)_) and > 15 Gy(RBE) (V_15 Gy(RBE)_), as well as the mean dose (D_mean_). To clearly predict the treatment beam path region, we also evaluated the total body volume receiving 10% of the prescribed dose outside the target and OAR regions. The formula for DVH loss_total_ is as follows:


(3) \begin{equation*} {\displaystyle \begin{array}{c}\mathrm{DVH}\ {\mathrm{loss}}_{\mathrm{CTV}}=\left|{\mathrm{D}}_{98\%}^{\mathrm{preDose}}-{\mathrm{D}}_{98\%}^{\mathrm{cliDose}}\right|+\left|{\mathrm{D}}_{2\%}^{\mathrm{preDose}}-{\mathrm{D}}_{2\%}^{\mathrm{cliDose}}\right|\end{array}} \end{equation*}

(4) \begin{align*} &\mathrm{DVH}\ {\mathrm{loss}}_{\mathrm{normal}\ \mathrm{liver}}=\left|{\mathrm{V}}_{30\ \mathrm{Gy}\left(\mathrm{RBE}\right)}^{\mathrm{preDose}}-{\mathrm{V}}_{30\ \mathrm{Gy}\left(\mathrm{RBE}\right)}^{\mathrm{cliDose}}\right|\nonumber\\&\quad+\left|{\mathrm{V}}_{15\ \mathrm{Gy}\left(\mathrm{RBE}\right)}^{\mathrm{preDose}}-{\mathrm{V}}_{15\ \mathrm{Gy}\left(\mathrm{RBE}\right)}^{\mathrm{cliDose}}\right|+\left|{\mathrm{D}}_{\mathrm{mean}}^{\mathrm{preDose}}-{\mathrm{D}}_{\mathrm{mean}}^{\mathrm{cliDose}}\right| \end{align*}

(5) \begin{equation*} {\displaystyle \begin{array}{c}\mathrm{DVH}\ {\mathrm{loss}}_{\mathrm{body}}=\left|{\mathrm{V}}_{10\%\mathrm{of}\ \mathrm{the}\ \mathrm{prescribed}\ \mathrm{dose}}^{\mathrm{preDose}}-{\mathrm{V}}_{10\%\mathrm{of}\ \mathrm{the}\ \mathrm{prescribed}\ \mathrm{dose}}^{\mathrm{cliDose}}\right|\end{array}} \end{equation*}

(6) \begin{equation*} {\displaystyle \begin{array}{c}\mathrm{DVH}\ {\mathrm{loss}}_{\mathrm{total}}=\mathrm{DVH}\ {\mathrm{loss}}_{\mathrm{CTV}}+\mathrm{DVH}\ {\mathrm{loss}}_{\mathrm{normal}\ \mathrm{liver}}+\mathrm{DVH}\ {\mathrm{loss}}_{\mathrm{body}}\end{array}} \end{equation*}

This loss function is designed to ensure that the predicted dose distribution adheres to clinical standards by minimizing errors in dose metrics across each target/OAR structure.

#### Dose gradient loss

In PBT, a characteristic dose gradient pattern is formed along the beam incidence direction. The dose gradient patterns in the lateral and distal fall-off regions, arising from the physical properties of the Bragg peak, represent clinically important spatial characteristics. However, conventional MSE loss, which is computed as an average over the entire dose distribution, cannot directly evaluate such local dose changes. Although gradient-based loss functions have been widely used in image processing, their application to dose distribution prediction in radiotherapy has not yet been reported, and no loss function has been proposed to explicitly learn dose gradient patterns specific to PBT. To address this limitation, we developed a dose gradient loss that computes direction-independent gradient magnitude in 3D dose distributions and evaluates the agreement of gradient patterns between clinical and predicted doses. Unlike general edge detection in image processing, this loss function was specifically designed to capture the physical characteristics inherent to PBT. By explicitly incorporating dose gradient information into the loss function, this approach enables the DL model to implicitly learn spatial characteristics associated with beam paths by focusing on the lateral and distal fall-off regions, without explicitly inputting beam arrangements. This enables prediction of physically reasonable dose distributions. The dose gradient loss is calculated based on the MSE between the gradient magnitudes of the predicted and clinical dose distributions. First, for each voxel, a direction-independent gradient magnitude $G(D)$ is computed from a dose distribution $D$ using the L2 norm. This is the square root of the sum of the squared gradients along the three patient coordinate axes (Right–Left, Anterior–Posterior, and Superior–Inferior). The formula for the dose gradient loss is as follows:


(7) \begin{equation*} {\displaystyle \begin{array}{c}\mathrm{dose}\ \mathrm{gradient}\ \mathrm{loss}=\frac{1}{\mathrm{N}}\sum_{\mathrm{i}=1}^{\mathrm{N}}{\left(G\left({preDose}_i\right)-G\left({cliDose}_i\right)\right)}^2\end{array}} \end{equation*}

where $G(D)$ is defined as follows:


(8) \begin{equation*} {\displaystyle \begin{array}{c}G(D)=\sqrt{{\left(\frac{\partial D}{\partial x}\right)}^2+{\left(\frac{\partial D}{\partial y}\right)}^2+{\left(\frac{\partial D}{\partial z}\right)}^2}\end{array}} \end{equation*}

and N is the total number of voxels. This loss function quantifies the agreement between the predicted and clinical dose distributions in terms of local dose changes.

The four types of combined loss functions were defined as follows:


(9) \begin{equation*} {\displaystyle \begin{array}{c}\mathrm{Loss}1=\mathrm{MSE}\ \mathrm{loss}\end{array}} \end{equation*}

(10) \begin{equation*} {\displaystyle \begin{array}{c}\mathrm{Loss}2=\mathrm{MSE}\ \mathrm{loss}+\mathrm{wMSE}\ \mathrm{loss}\end{array}} \end{equation*}

(11) \begin{equation*} {\displaystyle \begin{array}{c}\mathrm{Loss}3=\mathrm{MSE}\ \mathrm{loss}+\mathrm{wMSE}\ \mathrm{loss}+\mathrm{DVH}\ {\mathrm{loss}}_{\mathrm{total}}\end{array}} \end{equation*}

(12) \begin{equation*} {\displaystyle \begin{array}{c}\mathrm{Loss}4=\mathrm{MSE}\ \mathrm{loss}+\mathrm{wMSE}\ \mathrm{loss}+\mathrm{dose}\ \mathrm{gradient}\ \mathrm{loss}\end{array}} \end{equation*}

While the DVH loss and the dose gradient loss each reflect important information about the dose distribution, they only capture limited spatial information on their own. The DVH loss is a volume-based metric, while the dose gradient loss only evaluates local gradient information. If these loss functions are used in isolation, it becomes difficult for the model to learn a physically plausible dose distribution shape, making the model prone to getting trapped in local minima and leading to unstable convergence. Therefore, we adopted a composite approach that adds DVH loss or dose gradient loss to Loss2, which comprehensively captures spatial dose distribution information including target/OAR structural information. The weights for each loss term were selected to ensure that no single component becomes overly dominant and that all terms result in loss values of a similar order of magnitude (MSE loss: weight = 1, wMSE loss: weight = 1, DVH loss_total_: weight = 1e-4, and dose gradient loss: weight = 0.1). Specifically, these weight values were empirically determined through preliminary experiments that assessed the typical magnitude of each loss component during training. This selection was made to balance the contributions of all loss terms while maintaining training stability by preventing vanishing or exploding gradients. To ensure a fair comparison across all DL models, identical weight values were uniformly applied throughout.

### Model training

**Fig. 1:**
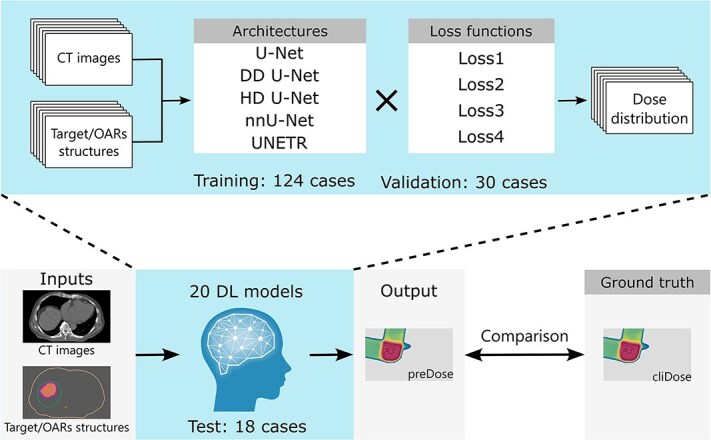
Overall workflow in the development of deep learning-based dose distribution prediction models for proton beam therapy with hepatocellular carcinoma patients.

The overall workflow for model development is presented in Fig. 1. The input data utilized for our DL models were treatment planning CT images and the structures of the CTV, PTV, spinal cord, liver, intestines, and body. To standardize input and output data across all cases, two normalization strategies were applied. CT values were rescaled to range between 0 and 1 using min-max normalization based on the typical HU range observed in the dataset. Dose distributions were normalized such that the dose delivered to 50% of the CTV was set to 1, regardless of the treatment protocol. This normalization process minimizes the influence of absolute prescription dose differences on the DL model’s learning, ensuring that the model learns relative spatial dose distribution patterns rather than absolute dose values. All normalization procedures were consistently applied to ensure a fair comparison across models.

Of the 172 available cases, 154 cases were assigned to the training dataset (training: 124 cases; validation: 30 cases) and 18 to the test dataset. Data splitting was performed using stratified random sampling to ensure that cases from both prescription dose groups were proportionally represented in each dataset. The training and validation dataset included 111 cases from the 66 Gy(RBE) group and 43 cases from the 64 Gy(RBE) group. The test dataset included 13 cases from the 66 Gy(RBE) group and 5 cases from the 64 Gy(RBE) group. Table 1 presents a detailed comparison of the training and test datasets, including the distribution of beam angles utilized in each. While certain beam angle regions exhibit notably low selection frequencies, the diversity of beam arrangements within our patient cohort is considered sufficient for the clinical context of PBT for HCC. Statistical analysis was performed with the Mann–Whitney U test, and no significant differences were found (*P* ≥ 0.01) between the dose metrics or beam angle usage frequencies of the training and test datasets, confirming the homogeneity of the two groups. A total of 20 DL models were developed by combining the five architectures with the four types of loss function. All 20 DL models were implemented in PyTorch 1.10.1 and trained on an NVIDIA RTX A6000 GPU. To ensure training stability and enable fair comparison across models, several strategies were adopted. Hyperparameter tuning for each DL model was performed using a grid search method. Learning rates (1e-5, 1e-4, 1e-3) and batch sizes (2, 4, 8) were explored, and the combination that yielded the lowest validation loss was selected. For all models, the learning rate was set to 1e-4 to minimize confounding factors in model comparison. The batch size was set to eight for the U-Net, DD U-Net, and nnU-Net models, two for the HD U-Net model, and four for the UNETR model. The Adam optimizer algorithm was used for model optimization, and the maximum number of epochs was set to 300. To prevent overfitting and improve training efficiency, early stopping was applied if the validation loss did not improve for 30 consecutive epochs. All other training parameters were kept consistent across all models. Training and validation loss curves were monitored throughout the training process for all 20 DL models. All models demonstrated stable convergence without significant oscillations or divergence. The gap between training and validation losses remained within acceptable limits across all models, confirming that severe overfitting did not occur.

**Table 1:** Comparison of patient and treatment plan characteristics between the training and test datasets used to develop deep learning-based dose distribution prediction models for proton beam therapy with hepatocellular carcinoma patients

		Training data	Test data	*P*-value
CTV (Mean ± SD)	Volume (cm^3^)	32.4 ± 33.8	24.6 ± 27.6	0.22
	D_98%_ (%)	97.7 ± 2.9	97.3 ± 2.6	0.90
	D_2%_ (%)	101.6 ± 0.9	101.9 ± 1.1	0.56
	CI	0.39 ± 0.11	0.38 ± 0.13	0.35
PTV (Mean ± SD)	Volume (cm^3^)	68.5 ± 55.2	54.6 ± 48.0	0.24
	D_98%_ (%)	89.0 ± 8.1	88.7 ± 7.4	0.89
	D_2%_ (%)	101.6 ± 0.8	101.8 ± 1.1	0.61
	CI	0.76 ± 0.07	0.76 ± 0.06	0.51
Normal liver (Mean ± SD)	D_mean_ (%)	8.0 ± 4.3	7.7 ± 4.1	1.00
	V_30 Gy(RBE)_ (%)	8.2 ± 4.6	7.9 ± 4.5	0.97
	V_<15 Gy(RBE)_ (cm^3^)	1165.4 ± 285.9	1116.3 ± 290.9	0.50
The percentage of utilized beam angles (%)	0°– 25°	10.8	16.6	0.27
	30°– 55°	0.6	0	1.00
	60°– 85°	0	0	N/A
	90°– 115°	0	0	N/A
	120°– 145°	0	0	N/A
	150°– 175°	9.1	8.3	1.00
	180°– 205°	23.4	27.8	0.54
	210°– 235°	4.9	11.1	0.13
	240°– 265°	7.1	2.8	0.49
	270°– 295°	33.8	27.8	0.58
	300°– 325°	5.8	5.6	1.00
	330°– 355°	4.5	0	0.38

All dose metrics are expressed as percentages relative to the prescribed dose.

CI, conformity index; N/A, not applicable

The trained DL models were used to predict 3D dose distributions using CT images and binary masks of the target/OAR structures of the test cases. The preDose distribution was subsequently normalized to ensure that the prescribed dose was delivered to 50% of the CTV.

Computational efficiency was evaluated by measuring both preprocessing and inference times. Preprocessing time included DICOM data loading, coordinate interpolation, RT structure parsing, mask processing, and tensor creation. Inference time for predicting a single 3D dose distribution was measured using a standardized benchmarking protocol. The mean preprocessing time per case was 98.8 ± 13.1 seconds (range: 65.2–121.8 seconds). The mean inference times were 37.5 ± 0.4 ms for U-Net, 37.1 ± 0.3 ms for DD U-Net, 178.1 ± 2.1 ms for HD U-Net, 74.0 ± 0.8 ms for nnU-Net, and 72.2 ± 0.4 ms for UNETR.

### Evaluation and analysis of the predicted dose distributions

The accuracy of the dose distribution predictions of each DL model was assessed by comparing the preDose and cliDose dose metrics. The dose differences between the two distributions were quantified using the mean errors and standard deviations (SDs).

Our evaluation metrics focused on clinically significant dose values for the target volumes and the normal liver. For target volume evaluation, D_98%_ and D_2%_ were calculated for both the CTV and PTV. In addition, the conformity index (CI) was calculated to evaluate the conformity of the dose distribution. The CI was calculated using Paddick’s formula, as shown in the following equation: [42]


(13) \begin{equation*} {\displaystyle \begin{array}{c}\mathrm{Conformity}\ \mathrm{index}=\frac{{\mathrm{TV}}_{\mathrm{PIV}}^2}{\mathrm{TV}\times \mathrm{PIV}}\end{array}} \end{equation*}

where treatment volume (TV) is the volume of the respective target, and the prescription isodose volume (PIV) is the total volume covered by the prescription dose in the target. The TV_PIV_ is the volume of overlap between the TV and PIV. The dose metrics for these target volumes are important in the evaluation of treatment plans [43].

The dose to the normal liver was evaluated using Dmean, V_30 Gy(RBE)_, and the volume receiving less than 15 Gy(RBE) (V_<15 Gy(RBE)_). These dose metrics were assessed as they are relevant to risk evaluation for radiation-induced liver disease [44–46]. As doses to the spinal cord and intestines were below 10% of the prescription dose in all cases, these structures were excluded from our quantitative evaluation. Statistical differences were identified using the Wilcoxon signed-rank test, with *P* < 0.01 considered statistically significant.

In our evaluation of the prediction accuracy of the beam path region in the preDose, we selected the 10% dose region as the target because it closely aligns with the beam path region. For each DL model, the evaluation was performed by calculating the mean absolute error (MAE) and the root mean squared error (RMSE) between the 10% dose region volumes derived from the cliDose and the preDose.

To further quantitatively assess the spatial agreement between the preDose and cliDose, the Dice similarity coefficient (DSC) was calculated for the 10% dose region across all test cases. As the proposed DL models predict dose distributions without explicitly inputting beam arrangements, accurate reproduction of this region demonstrates the model’s capacity to implicitly learn beam path characteristics. The DSC was computed as follows:


(14) \begin{equation*} {\displaystyle \begin{array}{c}\mathrm{DSC}=\frac{2\left|\mathrm{A}\bigcap \mathrm{B}\right|}{\left(\left|\mathrm{A}\right|+\left|\mathrm{B}\right|\right)}\end{array}}\end{equation*}

where A denotes the voxel set of the 10% dose region in the cliDose, and B denotes the corresponding region in the preDose. DSC values range from 0 to 1, where higher values indicate greater spatial concordance.

Additionally, DVH comparisons were conducted for two representative cases with different tumor sizes.

## RESULTS

### Dose metrics

The means and SDs of the cliDose and preDose dose metrics in test cases are summarized in Table 2. The preDose showed a mean D_98%_ for the CTV 3.3% lower than that of the cliDose (range: 77.6–98.3%). Conversely, the mean D_2%_ for the CTV was 2.5% higher than that of the cliDose (101.2–113.3%). Similarly, for the PTV, the preDose exhibited a mean D_98%_ that was 2.0% lower (75.0–92.1%) and a mean D_2%_ that was 2.6% higher (101.2–117.6%) than those of the cliDose. The preDose mean CI value was 0.07 higher for the CTV (0.27–0.54) and 0.03 lower for the PTV (0.58–0.81) than the cliDose values. The preDose D_mean_ for the normal liver was 0.3% lower than that of the cliDose (5.8–8.3%). The mean V_30 Gy(RBE)_ was 0.4% lower (6.0–8.1%), and the mean V_<15 Gy(RBE)_ was 2.2 cm^3^ higher (1101.7–1150.2 cm^3^) for the preDose than the cliDose.

**Table 2:** Summary of the preDose and cliDose dose metrics across all hepatocellular carcinoma patients treated with proton beam therapy in test cases. Data are shown as mean ± SD

Architecture	Loss function	CTV			PTV			Normal liver		
		D_98%_ (%)	D_2%_ (%)	CI	D_98%_ (%)	D_2%_ (%)	CI	D_mean_ (%)	V_30 Gy(RBE)_ (%)	V_<15 Gy(RBE)_ (cm^3^)
cliDose		97.3 ± 2.6	101.9 ± 1.1	0.38 ± 0.13	88.7 ± 7.4	101.8 ± 1.1	0.76 ± 0.06	7.7 ± 4.1	7.9 ± 4.5	1116.3 ± 290.9
U-Net	Loss1	90.1 ± 2.5^*^	113.3 ± 2.4^*^	0.27 ± 0.06^*^	88.1 ± 3.8	117.6 ± 3.6^*^	0.65 ± 0.07^*^	7.8 ± 3.8	7.9 ± 3.9	1118.1 ± 284.6
	Loss2	93.8 ± 3.6^*^	106.3 ± 3.9^*^	0.49 ± 0.09^*^	86.4 ± 2.4	106.0 ± 3.9^*^	0.71 ± 0.08	7.7 ± 3.9	8.0 ± 4.2	1114.9 ± 289.8
	Loss3	97.1 ± 2.4	102.3 ± 0.9	0.41 ± 0.12^*^	90.5 ± 1.5	102.4 ± 1.0^*^	0.81 ± 0.03	8.1 ± 3.9	7.8 ± 4.0	1104.7 ± 291.4
	Loss4	96.3 ± 2.0	102.8 ± 1.0	0.45 ± 0.10^*^	89.8 ± 1.4	102.6 ± 0.9	0.80 ± 0.06	7.5 ± 3.6	7.3 ± 3.6	1112.7 ± 286.5
DD U-Net	Loss1	77.6 ± 19.1^*^	110.3 ± 6.0^*^	0.44 ± 0.12	75.0 ± 14.1^*^	109.4 ± 6.6^*^	0.58 ± 0.06^*^	7.5 ± 3.7	7.2 ± 3.7	1112.2 ± 288.6
	Loss2	95.2 ± 3.0^*^	103.2 ± 3.1^*^	0.49 ± 0.09^*^	85.6 ± 4.1	103.6 ± 3.2^*^	0.71 ± 0.10	7.4 ± 3.5	7.4 ± 3.7	1118.0 ± 288.6
	Loss3	94.1 ± 6.9^*^	105.7 ± 2.7^*^	0.53 ± 0.07^*^	82.5 ± 3.2	105.6 ± 2.8^*^	0.66 ± 0.06^*^	5.8 ± 3.2^*^	6.0 ± 3.5^*^	1150.2 ± 282.1^*^
	Loss4	95.8 ± 2.4^*^	104.1 ± 2.0^*^	0.46 ± 0.12^*^	87.0 ± 2.7	104.1 ± 2.0^*^	0.72 ± 0.06	7.2 ± 3.4	7.2 ± 3.5	1122.5 ± 285.4
HD U-Net	Loss1	94.6 ± 3.7^*^	105.4 ± 2.1^*^	0.40 ± 0.09	89.3 ± 3.0	105.8 ± 2.5^*^	0.78 ± 0.06	7.9 ± 3.7	7.8 ± 3.8	1110.0 ± 290.4
	Loss2	95.8 ± 3.6	103.7 ± 1.8^*^	0.46 ± 0.08^*^	88.1 ± 2.1	103.4 ± 1.9^*^	0.75 ± 0.07	7.5 ± 3.8	7.4 ± 3.9	1117.1 ± 290.8
	Loss3	95.3 ± 5.2	103.1 ± 3.8	0.54 ± 0.08^*^	85.2 ± 3.0	103.1 ± 3.9	0.67 ± 0.10^*^	6.2 ± 3.3^*^	6.6 ± 3.6^*^	1142.8 ± 285.7^*^
	Loss4	98.3 ± 2.1	101.2 ± 0.7^*^	0.38 ± 0.14	92.1 ± 2.0	101.2 ± 0.7^*^	0.81 ± 0.04^*^	8.3 ± 3.9	8.1 ± 4.0	1101.7 ± 292.6
nnU-Net	Loss1	95.1 ± 3.6^*^	103.7 ± 1.7^*^	0.50 ± 0.08^*^	85.9 ± 3.1	103.4 ± 1.8^*^	0.70 ± 0.09	7.2 ± 3.5	7.2 ± 3.7	1120.5 ± 281.2
	Loss2	95.3 ± 2.6^*^	105.0 ± 1.2^*^	0.42 ± 0.13^*^	89.5 ± 3.1	105.1 ± 1.2^*^	0.75 ± 0.07	7.6 ± 3.4	7.8 ± 3.7	1117.1 ± 284.3
	Loss3	96.6 ± 1.9	102.5 ± 1.4	0.41 ± 0.12	89.4 ± 3.3	102.8 ± 1.4	0.75 ± 0.06	7.8 ± 3.6	7.8 ± 3.7	1111.3 ± 280.2
	Loss4	97.2 ± 1.7	101.9 ± 1.1	0.44 ± 0.13^*^	89.5 ± 2.7	102.1 ± 1.2	0.77 ± 0.06	7.6 ± 3.7	7.6 ± 3.8	1115.4 ± 283.0
UNETR	Loss1	94.4 ± 3.9^*^	104.0 ± 2.0^*^	0.46 ± 0.13^*^	86.4 ± 2.4	103.5 ± 2.1^*^	0.68 ± 0.12	7.4 ± 3.3	7.5 ± 3.5	1120.0 ± 282.1
	Loss2	95.4 ± 5.7	103.2 ± 1.7^*^	0.43 ± 0.09^*^	88.6 ± 4.3	102.8 ± 1.7	0.77 ± 0.08	7.5 ± 3.5	7.6 ± 3.6	1117.7 ± 286.2
	Loss3	94.5 ± 4.0^*^	102.8 ± 1.9	0.50 ± 0.09^*^	82.5 ± 4.7	102.5 ± 1.9	0.66 ± 0.11^*^	7.3 ± 3.4	7.4 ± 3.6	1119.0 ± 283.3
	Loss4	94.7 ± 7.8	102.8 ± 1.4	0.43 ± 0.09^*^	87.1 ± 6.2	102.4 ± 1.5	0.76 ± 0.10	7.3 ± 3.4	7.4 ± 3.6	1123.9 ± 284.3

Asterisks indicate a significant difference from cliDose (^*^P < 0.01). All dose metrics are expressed as percentages relative to the prescribed dose.

cliDose, clinical dose; preDose, predicted dose; SD, standard deviation

**Fig. 2:**
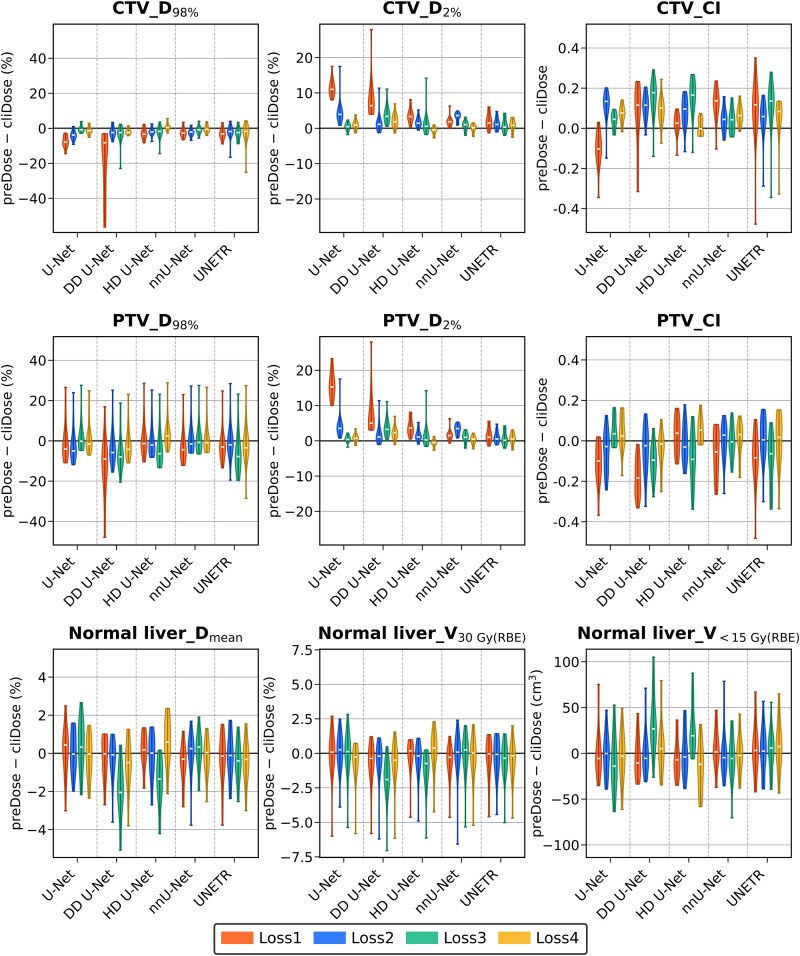
Dose metric differences (preDose − cliDose) for the CTV, PTV, and normal liver across the different network architectures and loss functions used to develop deep learning models for the prediction of dose distribution in proton beam therapy. These violin plots illustrate the differences between the predicted and clinical doses for deep learning models. Positive values indicate overprediction and negative values indicate underprediction. The white lines denote the median values, and the width of each violin reflects the distribution of the differences. cliDose, clinical dose; CTV, clinical target volume; preDose, predicted dose; PTV, planning target volume.

The differences in dose metrics between the preDose and cliDose (preDose − cliDose) for the CTV, PTV, and normal liver are summarized in Fig. 2. For the target volumes, DL models using Loss1 tended to underestimate D_2%_ and overestimate D_98%_ for both the CTV and PTV, regardless of the architecture. In contrast, DL models using Loss4 demonstrated smaller prediction errors than the models using the other loss functions. DL models using Loss3 exhibited larger deviations in the dose evaluation metrics for the normal liver than other models, with particularly notable trends observed in DD U-Net and HD U-Net, where D_mean_ tended to be underestimated and V_<15 Gy(RBE)_ tended to be overestimated. The DL model combining nnU-Net with Loss4 showed median differences close to zero for dose metrics related to the normal liver. For the UNETR models, the errors in the CI for the CTV and PTV exhibited a large variance, regardless of the loss function.

### Isodose volume

**Fig. 3:**
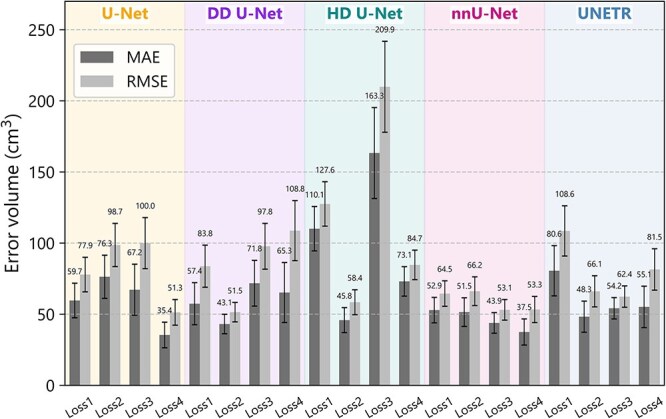
MAEs and RMSEs for 10% dose region between cliDose and preDose across DL models developed for the prediction of dose distribution in proton beam therapy. Error bars represent standard error of the mean, calculated as standard deviation divided by the square root of the sample size (*n* = 18). cliDose, clinical dose; DL, deep learning; MAE, mean absolute error; preDose, predicted dose; RMSE, root mean squared error.

The MAE and RMSE of the 10% dose region volumes between cliDose and the preDose of each DL model are presented in Fig. 3. Across all models, the MAE values ranged from 35.4 cm^3^ to 163.3 cm^3^, and the RMSE values ranged from 51.3 cm^3^ to 209.9 cm^3^. The DL model that combined U-Net architecture with Loss4 achieved the smallest errors, with an MAE of 35.4 cm^3^ and an RMSE of 51.3 cm^3^. These results indicate that incorporating dose gradient information into the loss function substantially improves the accuracy of beam path shape prediction, which is critical for evaluating dose delivery to normal tissues along the beam path. Among the architectures, the nnU-Net models demonstrated the smallest error volumes, with a mean MAE of 46.4 cm^3^ and a mean RMSE of 59.3 cm^3^.

### Visual analysis of the predicted dose distributions

**Fig. 4:**
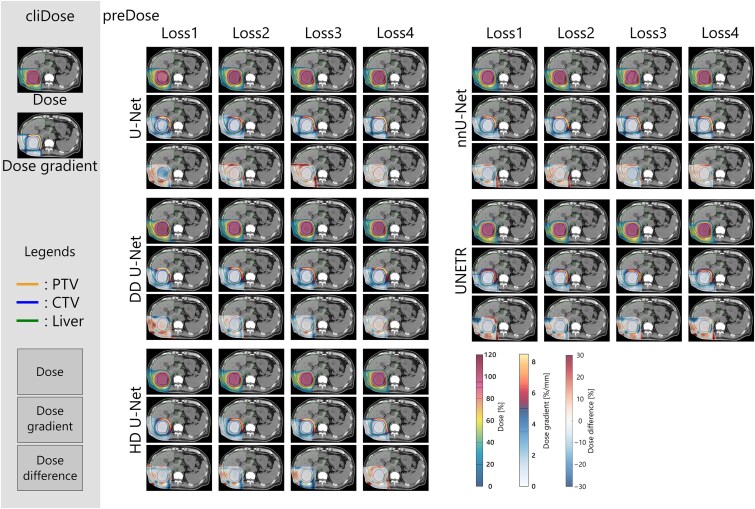
The doses, dose gradients, and dose difference distributions for a representative case from 20 deep learning models developed for the prediction of dose distribution in proton beam therapy.

Figure 4 shows the predicted dose distributions, dose gradient distributions, and dose difference distributions (preDose−cliDose) from each DL model for a representative case. When using Loss1, scattered, non-uniform dose prediction errors were observed across all architectures. In contrast, with Loss2, prominent dose prediction errors were observed in regions without ROIs, and the predicted dose distributions tended to expand concentrically around the CTV and PTV. Using Loss3, dose prediction errors were notable in the beam lateral fall-off regions, and the dose gradients in the distal fall-off regions were more gradual than those of the cliDose. With Loss4, dose prediction errors in both the beam lateral and distal fall-off regions were lower across all network architectures, and the overall dose distribution shapes closely resembled those of the cliDose. U-Net and nnU-Net demonstrated the smallest dose prediction errors in the beam lateral and distal fall-off regions, particularly when combined with Loss4. In the DD U-Net models, dose distributions tended to expand concentrically around the CTV and PTV, regardless of the loss function used. HD U-Net consistently showed smaller dose prediction errors within the CTV across all loss functions. The UNETR models, regardless of the loss function employed, tended to overestimate the dose in the proximal plateau region and exhibited lateral dose errors. In contrast, nnU-Net, particularly when using Loss4, produced predicted doses that were in close agreement with the clinical dose, resulting in minimal dose discrepancies within both the CTV and PTV. These visual results directly support the hypothesis that dose gradient loss functions can effectively capture spatial characteristics related to beam paths. Specifically, dose distributions predicted by DL models with Loss4 showed sharp transitions at the lateral and distal fall-off regions, closely resembling the dose gradient patterns of the cliDose. By contrast, DL models trained with other loss functions tended to generate more gradual or spatially offset dose gradients in these regions. Notably, U-Net and nnU-Net, particularly when combined with Loss4, exhibited the smallest dose prediction errors in these clinically significant fall-off regions.

### Spatial agreement

**Fig. 5:**
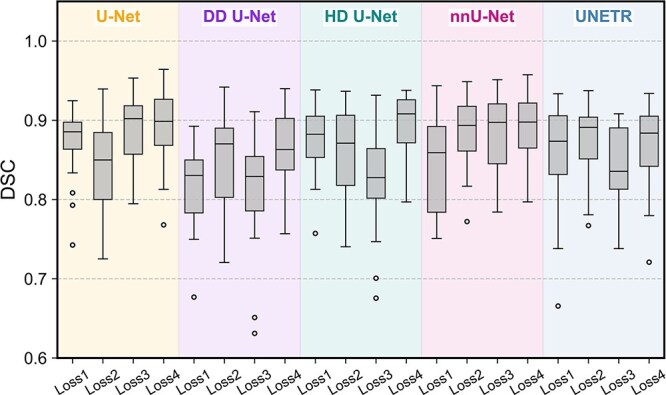
Spatial agreement of 10% dose region evaluated using Dice similarity coefficient. DSC values for the 10% dose region between cliDose and preDose across all DL models in the test dataset (*n* = 18). DSC, Dice similarity coefficient; cliDose, clinical dose; preDose, predicted dose; DL, deep learning.

Figure 5 presents the DSC values for the 10% dose region between cliDose and preDose across all DL models. Across all 20 model combinations, the mean DSC values ranged from 0.81 to 0.90. Among the evaluated architectures, nnU-Net achieved the highest DSC (0.88 ± 0.05), followed by U-Net (0.87 ± 0.05). Among the loss functions, Loss4 achieved the highest DSC (0.88 ± 0.05), followed by Loss2 (0.86 ± 0.06). Models using Loss4 consistently demonstrated higher mean DSC values compared to those using Loss2 across all architectures except UNETR.

### DVH analysis

**Fig. 6:**
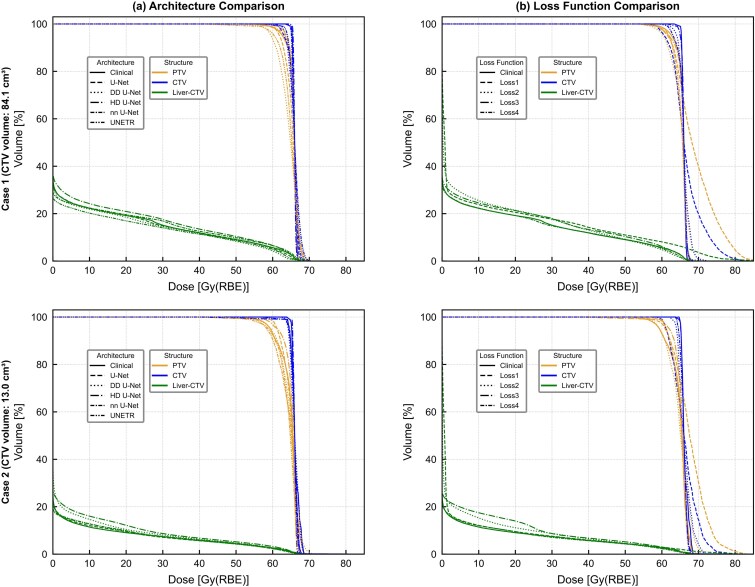
Dose–volume histogram comparison for two representative cases with different tumor sizes. Case 1 represents a large target (CTV volume: 84.1 cm^3^) and Case 2 represents a small target (CTV volume: 13.0 cm^3^). (a) Comparison of five network architectures with the loss function fixed to Loss4. (b) Comparison of four loss functions with the architecture fixed to U-Net. Solid lines indicate clinical dose, and dashed lines indicate predicted dose from each model. CTV, clinical target volume; DVH, dose–volume histogram; PTV, planning target volume.

To provide visual insight into the dose prediction accuracy across the entire dose–volume relationship, DVH comparisons were performed for two representative cases with different tumor sizes: a large tumor case (CTV volume: 84.1 cm^3^) and a small tumor case (CTV volume: 13.0 cm^3^). Figure 6 shows the DVH curves for the CTV, PTV, and normal liver, comparing both the effect of network architecture under a fixed loss function (Loss4) and the effect of loss function under a fixed architecture (U-Net).

In both cases, the DVH curves of the preDose predicted by U-Net and nnU-Net using Loss4 showed the best agreement with the cliDose across all evaluated target/OAR structures. For the target structures, these models accurately reproduced the steep dose gradients in the high-dose regions, with minimal deviations from the cliDose DVH. For the normal liver, the predicted DVH curves maintained good agreement in the low- to intermediate-dose regions, which are important for risk evaluation of radiation-induced liver disease. In contrast, models using Loss3 exhibited notable deviations in the low-dose regions of the normal liver DVH. These findings are consistent with the quantitative results presented in the preceding sections, further supporting the conclusion that loss functions incorporating dose gradient information improve prediction accuracy in low-dose regions, which are closely related to the beam path regions and clinically important.

## DISCUSSION

We developed several DL models for dose distribution prediction in PBT for HCC. We compared their prediction accuracy across various network architecture and loss function combinations. DL models that combine simple architectures like U-Net or nnU-Net with a physics-informed, dose gradient-aware loss function predicted the dose distribution that approximated the cliDose in PBT for HCC.

The two novel loss functions we developed in this study produced contrasting results in their dose distribution prediction accuracy. Despite being directly related to the clinical evaluation metric, the loss function that incorporated the DVH evaluation metric tended to be less accurate, especially in dose predictions for the normal liver. This may be because DVH loss is a volume-based assessment, so fails to capture the local characteristics of the spatial dose distribution. The dose gradient becomes steep in the region immediately outside the target due to the Bragg peak, and it appears that a loss function that uses the target ROI cannot adequately evaluate the dose distribution characteristics of PBT. On the other hand, the use of a loss function that incorporates the dose gradient improved dose distribution prediction accuracy, particularly in clinically important regions such as the lateral and distal fall-off regions in PBT. A comprehensive evaluation of our results reveals that Loss4, compared to other loss functions, enables more flexible dose distribution prediction from only CT images and target/OAR structures, without requiring a predefined beam arrangement, and produces the most accurate preDose that most closely approximates the cliDose. The comprehensive evaluation presented in this study strongly suggests the importance of gradient-aware learning in PBT dose prediction. The superiority of Loss4 was consistently confirmed across all evaluation metrics. Specifically, dose metrics for the target volumes demonstrated reduced prediction errors; MAE for the 10% dose region was minimized; DSC values for isodose volume overlap achieved the highest scores; and DVH curve agreement was best maintained across all structures. Crucially, the visual analysis revealed that Loss4 models accurately reproduced the steep dose gradients at the lateral and distal fall-off regions, which are the most critical areas for distinguishing between therapeutic dose delivery and normal tissue sparing in PBT. The gradient-aware loss function enabled accurate reproduction of the dose distribution shape arising from the Bragg peak by directly targeting these regions of steep dose change during training. By explicitly incorporating these physical characteristics of proton beams into the loss function, our approach serves as a physics-informed learning framework that captures the dose distribution shape patterns specific to PBT. Specifically, the gradient magnitude reflects the local rate of dose change: high gradient values are observed perpendicular to the beam incidence direction, while relatively low gradient values persist along the beam path until the Bragg peak is reached. By learning this gradient pattern, the DL model was able to indirectly infer clinically plausible beam paths without explicitly inputting beam arrangements. This convergent evidence across multiple independent evaluation metrics strongly supports the conclusion that dose gradient information is essential for accurate PBT dose prediction, and that conventional loss functions that do not explicitly account for dose gradient patterns are insufficient to capture the dosimetric characteristics unique to PBT.

Regarding the network architecture, when using Loss4, basic U-Net and nnU-Net demonstrated dose distribution predictions closer to the cliDose than those resulting from more complex architectures such as DD U-Net, HD U-Net, and UNETR. This finding contrasts somewhat with previous reports in which DD U-Net and HD U-Net achieved superior dose prediction performance in intensity-modulated radiation therapy (IMRT) for head and neck cancers [35, 36]. This discrepancy may be attributed to the uniformity of the tissue surrounding the target in HCC and the dose distribution characteristics specific to PBT. In this study, HCC was targeted, in which the surrounding tissue is predominantly hepatic, with relatively homogeneous tissue density. Therefore, in this context, even U-Net was sufficient to extract the necessary features for accurate dose prediction. Previous studies have shown that DD U-Net and HD U-Net are well-suited to dose distribution prediction for target areas with complex anatomical structures and varying tissue densities, such as the bone and soft tissue in the head and neck region. However, in more homogeneous structures such as the liver and surrounding area, complex network architectures may not have contributed significantly to improved prediction accuracy. Additionally, while the dose distribution in IMRT typically exhibits a circular pattern, with concentric dose levels centered around the target, the dose distribution in PBT is highly dependent on the direction of beam incidence. Therefore, the simpler architecture of U-Net performed more effectively in dose distribution prediction for PBT. Nevertheless, when we used the same architecture and loss function settings as the previous studies mentioned above, we achieved smaller prediction dose errors for the CTV and PTV with DD U-Net and HD U-Net than with U-Net. Furthermore, the prediction errors in the low- to intermediate-dose regions reported in those studies were confirmed in our DL models using the same settings. The nnU-Net model demonstrated more stable dose prediction accuracy compared to the UNETR model. This finding aligns with other reports comparing these two architectures, which have similarly highlighted superior prediction accuracy for target volumes such as the CTV and PTV [47]. For a moderately-sized dataset like ours (*n* = 172), these results suggest that the strong inductive bias inherent in CNNs may be more advantageous than the theoretical flexibility of Transformers, a conclusion supported by the previous study [47].

We focused on treatment plans using the PS method. The PS method employed in this study and the pencil beam scanning (PBS) method, now widely used in clinical practice, exhibit notable physical and dosimetric differences in their beam characteristics, delivery techniques, and treatment planning optimization processes. Consequently, a direct application of the DL model constructed from PS data to PBS dose prediction is fundamentally challenging. However, the methodology established in this study for constructing the DL model is considered to possess a universality that is not specific to any particular irradiation technique. Therefore, by training a new model from scratch with a PBS case dataset, we anticipate that high-precision dose distribution prediction can also be achieved for PBS. Furthermore, as a more efficient approach, the application of transfer learning can be considered. Previous research has demonstrated that a model pre-trained on PS data, when fine-tuned with a small number of PBS cases, can achieve high generalization performance for intensity-modulated PBT data from different institutions [48]. This finding suggests that our model possesses potential for application to PBS through a similar approach.

Previous studies have demonstrated that beam mask-based methods can achieve high prediction accuracy by explicitly incorporating beam arrangement information into the model input [26, 29]. However, while such approaches may be effective when the optimal beam arrangement is already known, they impose constraints on clinical flexibility by requiring predetermined beam arrangements. Conversely, the beam arrangement-free framework proposed in this study directly predicts dose distributions from only CT images and target/OAR structures, eliminating the need for prior beam arrangement preparation. The DL models developed in this study, particularly the combination of simple architectures like U-Net or nnU-Net and Loss4, demonstrated the ability to predict dose distributions close to the cliDose directly from the patient’s CT images and target/OAR structures without the need for preparations such as beam arrangement. A previous study that conducted a comprehensive evaluation using the largest clinical dataset for head and neck PBT dose prediction (*n* = 632) reported that for cases with unilaterally localized targets, DSC values at the 10% isodose level were ~0.84 without beam masks and ~0.90 with beam masks [29]. In comparison, our proposed method achieved a DSC value of 0.88 ± 0.05 (nnU-Net with Loss4). This performance is comparable to that of the beam mask-based model in the previous study, demonstrating that highly accurate predictions are achievable without explicit beam arrangement information. However, DSC values for the 10% dose region are influenced by several case-specific factors. Because the 10% isodose volume spans the entire beam path, its shape depends on tumor size and location. Larger or deeper-seated tumors produce broader beam paths and larger 10% isodose volumes, increasing spatial overlap and yielding higher DSC values. Conversely, smaller or shallower tumors result in narrower beam paths, making DSC values more sensitive to minor spatial discrepancies. These factors should be considered when interpreting DSC values across different patient anatomies. This approach is positioned as a critical preliminary step toward the full automation of treatment planning in PBT. Our findings provide immediate clinical value from multiple perspectives. Specifically, using the preDose as a guideline tool or an optimization objective for planners is expected to equalize plan quality, reduce inter-planner variability, and shorten the overall planning time. Furthermore, our DL model enables medical institutions lacking PBT facilities or expertise to virtually evaluate patient-specific PBT dose distributions, thereby supporting informed decision-making regarding PBT indication. These capabilities are expected to lower the technical barrier for physicians in assessing the potential benefits of PBT on a patient-by-patient basis, contributing to the expansion of patient access to PBT. It should be noted that the proposed method is intended to support treatment planning assistance in the planning process and clinical decision-making regarding treatment options, rather than to replace inverse planning optimization.

This study has several limitations. First, the DL model developed herein is limited to the prediction of a 3D dose distribution. Integrating these predictions into the clinical workflow requires the development of technology for the inverse calculation of necessary parameters to create a deliverable treatment plan, as demonstrated in previous research [24, 25]. This remains an important subject for future research. Second, this study used the pencil beam algorithm for dose calculation. The Monte Carlo (MC) algorithm enables precise dose calculations in PBT planning [49, 50]. In many cases within the patient cohort of this study, dose calculations were performed within the homogeneous liver, suggesting that the difference in dose calculation accuracy is within a practically acceptable range. However, in some cases where the tumor was located at the interface with the diaphragm, the impact of dose calculation errors may be relatively larger, especially for smaller target sizes [51, 52]. Loss4, as presented in this study, may be particularly well-suited to capturing the complexity of MC-calculated dose distributions. However, further research is needed to provide comprehensive validation of this application. Third, a limitation relates to the composition of the patient cohort. This study included only cases in which the distance between the tumor and OARs such as portal vein branches and the gastrointestinal tract was 20 mm or greater; consequently, cases with OARs in close proximity to the target were not included in the dataset. For example, in cases of tumors adjacent to the gastrointestinal tract, beam entry angles are often severely constrained to avoid direct irradiation of radiosensitive structures, resulting in dose distributions with limited directional diversity. Similarly, tumors located near the porta hepatis may require beam paths that carefully avoid irradiating these critical structures, producing complex dose gradient patterns [53]. In such beam-arrangement-constrained scenarios, dose distributions may differ substantially from those observed in the present cohort, and the prediction accuracy of the proposed DL model remains unverified. Within the scope of this study, doses to the spinal cord and intestines were less than 10% of the prescription dose in all cases, suggesting that the DL model appropriately reproduced the low-dose regions associated with beam path areas. However, this result does not guarantee generalizability to cases with OARs in close proximity to the target. Future studies should investigate the applicability of the proposed method to more complex scenarios by including cases with OARs adjacent to the target under different treatment protocols. Furthermore, training the DL model using multi-institutional datasets is expected to encompass a wider variety of cases and beam arrangement strategies, thereby further improving the generalizability and robustness of the model.

In summary, this study systematically demonstrated that dose gradient-aware learning is essential for improving the accuracy in dose distribution prediction for PBT for HCC. The proposed dose gradient loss function explicitly targets the steep lateral and distal fall-off regions characteristic of PBT, enabling the reproduction of spatial characteristics that closely approximate clinical dose distributions without explicitly inputting beam arrangements. The convergent results across multiple independent evaluation metrics indicate that the physics-informed, dose gradient-aware loss function can effectively learn proton-specific dose gradient patterns that conventional loss functions fail to capture, establishing that such learning is critical for accurate PBT dose distribution prediction. The clinical significance of this method lies in its potential to serve as a guideline tool that provides treatment planners with immediately accessible dose distributions at an early stage of the treatment planning workflow. Additionally, it holds potential value in supporting clinical decision-making regarding treatment options by enabling medical institutions lacking PBT facilities or expertise to virtually evaluate patient-specific dose distributions. However, the findings of this study are based on a single-institutional dataset, and the applicability to complex cases where OARs are in close proximity to the target or to different irradiation techniques remains unverified. Therefore, the present results should be regarded as a first step in demonstrating the importance of dose gradient-aware learning in proton dose prediction. For clinical implementation, validation of external validity using multi-institutional data and the development of inverse calculation technology to create deliverable treatment plans remain as future challenges.
